# Rubella seroprevalence among pregnant women in Beijing, China

**DOI:** 10.1186/s12879-018-3032-x

**Published:** 2018-03-15

**Authors:** Qinghong Meng, Jie Luo, Lijun Li, Wei Shi, Jinqian Yu, Yingjie Shen, Li Li, Yajuan Wang, Kaihu Yao

**Affiliations:** 1 Beijing Key Laboratory of Pediatric Respiratory Infection Diseases, Key Laboratory of Major Diseases in Children, Ministry of Education, National Clinical Research Center for Respiratory Diseases, National Key Discipline of Pediatrics (Capital Medical University), Beijing Pediatric Research Institute, Beijing Children’s Hospital, Capital Medical University, National Center for Children’s Health, Beijing, China; 2 Department of Neonatology, Beijing Children’s Hospital, Capital Medical University, National Center for Children’s Health, Beijing, China; 3 Department of Neonatology, Shunyi Women and Children’s Hospital of Beijing Children’s Hospital, Beijing, China

**Keywords:** Rubella, Antibodies, Pregnant women, Rubella immunization

## Abstract

**Background:**

Rubella infection in pregnant women can result in serious effects, such as miscarriages, stillbirths, and congenital rubella syndrome (CRS). However, very little is known about the rubella seroprevalence among pregnant women in China.

**Methods:**

This is a cross-sectional and hospital-based study. From June 2016 through March 2017, a total of 324 serum samples from healthy pregnant women were collected in the Shunyi Women and Children’s Hospital of Beijing Children’s Hospital. Rubella-specific IgG antibody was determined by ELISA (Euroimmun, Lübeck, Germany) kits. International assigned cut-off values of ≥10 IU/ml were used to assess the percentage of pregnant women with protective IgG concentrations.

**Results:**

The total rate of protected individuals was 83.3% (95% CI: 78.9%–87.0%). The protective rates of pregnant women in 17–26 years group, 27–36 years group and 37–46 years group were 84.0% (95% CI: 75.3%–90.1%), 81.9% (95% CI: 74.9%–87.4%) and 84.9% (95% CI: 75.8%–90.9%) respectively. No significant difference in protective rates among the three age groups was found (*P* = 0.83). There were also no statistically significant correlations between protective rates and gravidity (*P* = 0.84), parity (*P* = 0.84), birth place (*P* = 0.16), residence area (*P* = 0.58), education (*P* = 0.40) or occupation (*P* = 0.65).

**Conclusions:**

Despite the generally low vaccination coverage for rubella, most of Chinese pregnant women had potent rubella immunity. However, at least 16.7% of pregnant women were susceptible to rubella, which suggested rubella immunization in Chinese women at or before child-bearing age.

## Background

Rubella, also known as German measles, is usually a relatively benign disease in children caused by *Rubella virus*. However, rubella infection in pregnant women, especially during the first trimester, can result in miscarriages, stillbirths, and congenital rubella syndrome (CRS), a constellation of birth defects that often includes cataracts, hearing loss, mental retardation, and congenital heart defects [1]. Recently, outbreaks of rubella have reemerged in some countries, such as Italy [2], Romania [3], Japan [4], and Tunisia [5].

There is no specific treatment for rubella and CRS, but they can be prevented by immunization. With the implementation of rubella vaccination strategies, endemic rubella transmission has been interrupted in the Americas since 2009 [6]. However, Japan and other countries have been confronted with rubella outbreaks because of the partial vaccination strategy [7, 8]. The MMR (measles-mumps-rubella) vaccine contains live, attenuated viruses for measles, mumps and rubella [9]. To avoid the theoretical risk for fetal complications, MMR vaccine was not administered for pregnant women. In the United States, the Advisory Committee on Immunization Practices (ACIP) recommended that non-pregnant women of child-bearing age without evidence of rubella immunity should receive 1 dose of MMR [10].

The present whole national immunization program was started from 1978 in China. Only four basic vaccines against six contagious diseases (tuberculosis, diphtheria, tetanus, whooping cough, poliomyelitis, and measles) were included [11]. It was not available to get rubella vaccine (the domestic BRDII and imported RA27/3 vaccines) until 1990s [12]. Meanwhile, the rubella vaccine coverage was low because it was included in private sector. The rubella vaccine was added into the national Expanded Program on Immunization of China in 2008 [11], in which the MR (measles-rubella) immunization is administrated at 8 months of age, and a booster immunization with MMR at 18–24 months of age [13]. Despite the efforts of vaccination, the epidemiology of rubella was not be well revealed by the current surveillance system. In Beijing and Shandong Province, a shift of peak incidence of age with rubella from young children to 15–39 year-old group has been found [13, 14]. According to the data from Jinan and Yantai in 2007, the rate of CRS was 9 cases per 1000 live births [15]. These reports suggested that the published national figures of rubella cases could not reflect the reality of rubella in China.

The main aims of vaccination programs are to prevent rubella infection during pregnancy and to protect CRS. The seroprevalence of rubella virus infection among pregnant women in several countries has been reported [16–21]. However, the rubella seroprevalence in Chinese pregnant women population has been scantily studied, only one report among Chinese women of reproductive age during preconception period were available to estimate the immune protection level and CRS risk in our country [22]. Therefore, we conducted a seroepidemiology study to assess the level of protective immunity and risk to rubella in pregnant women in China.

## Method

### Samples collection

This is a cross-sectional and hospital-based study. From June 2016 through March 2017, 324 pregnant women who were about to deliver their baby in the Shunyi Women and Children’s Hospital of Beijing Children’s Hospital were included in this study. The cases with immune system related diseases and other chronic medical conditions (diabetes, hypertension, liver and kidney diseases) were not enrolled.

A recent seroepidemiological study conducted in our country reported that the overall prevalence of anti-rubella IgG seroprevalence for women at childbearing age in Beijing was 91.9% [22]. Studies on seroprevalence of anti-rubella IgG conducted in other countries have reported that the seroprevalence in pregnant women varied from 85 to 95% [16, 20, 23]. Assuming conservatively a positive rate of 80%, a significance level of 5%, a minimum number of 316 samples was needed for pregnant women respectively to achieve 80% power.

After obtaining informed consent, a routinely maternal blood sample was obtained at 35 weeks of gestational age. All serum samples were frozen at − 20°C until analysis. Information about subject’s age or date of birth, gestational age, birth place (Beijing or other city), residence area (urban or rural), gravidity (one or more), parity (one delivery or more), education (Non-educated/Primary school/Junior high school/Senior high school/College or higher) and occupation (Unemployed/Employed) was also collected to assess the possible effect of rubella on the fetus.

### Serological testing

Anti-rubella IgG was detected using the commercially available ELISA kit (Euroimmun, Lübeck, Germany), according to the manufacturer’s instructions. The antibody results were expressed in international units per mili-liter (IU/ml). The lower limit of detection was 1 IU/ml. The anti-rubella IgG levels were categorized as negative, equivocal and positive when values obtained were < 8, 8- < 11 and ≥11 IU/ml, respectively, according to the manufacturer’s protocol. International assigned cut-off values of ≥10 IU/ml were used to assess the percentage of pregnant women with protective IgG concentrations, according to previous reports [24, 25].

### Data analysis

Data were analyzed using the GraphPad Prism software (version 5; GraphPad Software, La Jolla, CA, USA) and JMP (version 10.0). χ^2^ test was used to compare proportions of subjects with protective anti-rubella IgG among different subgroup. *P* ≤ 0.05 were considered statistically significant.

## Results

A total of 324 pregnant women who were to deliver their babies in the Shunyi Women and Children’s Hospital of Beijing Children’s Hospital were asked to participate in the study. The mean maternal age at the time of delivery was 30.5 years (21–46 years). Their gravidity ranged from 1 to 8, and the parity ranged from 1 to 3. It was the first pregnancy for 39.5% of the women. Of the 324 women, 50.6% were born in Beijing, and 40.7% lived in urban areas. About 61.4% of the pregnant women had a college education or higher.

Of the 324 tested pregnant women, anti-rubella IgG concentration below the lower limit of detection occurred in only 16 (4.9%; 95% CI: 3.1%–7.9%) cases. The proportions of negative, equivocal and positive anti-rubella IgG were 11.7% (95% CI: 8.7%–15.7%), 5.9% (95% CI: 3.8%–9.0%) and 82.4% (95% CI: 77.9%–86.1%), respectively. Protective concentrations of anti-rubella ≥10 IU/ml were seen in 83.3% (95% CI: 78.9%–87.0%) of serum samples (Table 1).

**Table 1:** Distributions of anti-rubella antibodies in pregnant women by age

		N, % (95% CI)				
	*N*	Negative (< 8 IU/ml)	equivocal (8- < 11 IU/ml)	Positive (≥11 IU/ml)	Protective (≥10 IU/ml)	*P*
Total	324	38 (11.7%; 8.7%–15.7%)	19 (5.9%; 3.8%–9.0%)	267 (82.4%; 77.9%–86.1%)	270 (83.3; 78.9%-87.0%)	
Age						
17-26y	94	11 (11.7%; 6.7%-19.6%)	5 (5.3%; 2.3%–11.9%)	78 (83.0%; 74.1%–89.2%)	79 (84.0%; 75.3%–90.1%)	0.83
27-36y	144	18 (12.5%; 8.1%- 18.9%)	9 (6.3%; 3.3%–11.5%)	117 (81.3%; 74.1%–86.8%)	118 (81.9%; 74.9%–87.4%)	
37-46y	86	9 (10.5%; 5.6%- 18.7%)	5 (5.8%; 2.5%–12.9%)	72 (83.7%; 74.5%–90.0%)	73 (84.9%; 75.8%–90.9%)	

The anti-rubella levels of pregnant women were also analyzed by different age group. Because the vaccination records of these pregnant women could not be obtained, they were divided into three groups according to birth date. Among them, 94 subjects aged 17–26 years who were born after 1990, and 144 subjects aged 27–36 years who were born between 1980 and 1990. Another 86 subjects aged 37–46 years who were born before 1980. The protective rates of pregnant women in 17–26 years group, 27–36 years group and 37–46 years group were 84.0% (95% CI: 75.3%–90.1%), 81.9% (95% CI: 74.9%–87.4%) and 84.9% (95% CI: 75.8%–90.9%) respectively. No significant difference in protective rates among the three age groups was found (*P* = 0.83) (Table 1). There were also no statistically significant correlations between protective rates and gravidity (*P* = 0.84), parity (P = 0.84), birth place (*P* = 0.16), residence area (*P* = 0.58), education (*P* = 0.40) or occupation (*P* = 0.65) (Tables 2 and 3).

**Table 2:** Distributions of anti-rubella antibodies in pregnant women by gravidity and parity

		N, % (95% CI)				
	*N*	Negative (< 8 IU/ml)	equivocal (8- < 11 IU/ml)	Positive (≥11 IU/ml)	Protective (≥10 IU/ml)	*P*
Gravidity						
One	128	16 (12.5%; 7.8%–19.3%)	8 (6.3%; 3.2%–11.8%)	104 (81.3%; 73.6%–87.1%)	106 (82.8%; 75.3%–88.4%)	0.84
More than one	196	22 (11.2%; 7.5%–16.4%)	11 (5.6%; 3.2%–9.8%)	163 (83.2%; 77.3%–87.8%)	164 (83.7%; 77.9%–88.2%)	
Parity						
One	194	22 (11.3%; 7.6%–16.6%)	13 (6.7%; 4.0%–11.1%)	159 (82.0%; 75.9%–86.7%)	161 (83.0%; 77.1%–87.6%)	0.84
More than one	130	16 (12.3%; 7.7%–19.1%)	6 (4.6%; 2.1%–9.7%)	108 (83.1%; 75.7%–88.6%)	109 (83.8%; 76.6%–89.2%)

**Table 3:** Distributions of anti-rubella antibodies in pregnant women by socio-demographic characteristics

		N, % (95% CI)				
	*N*	Negative (< 8 IU/ml)	equivocal (8- < 11 IU/ml)	Positive (≥11 IU/ml)	Protective (≥10 IU/ml)	*P*
Birth place						
Beijing	164	23 (14.0%; 9.5%–20.2%)	10 (6.1%; 3.3%–10.9%)	131 (79.9%; 73.1%–85.3%)	132 (80.5%; 73.8%–85.8%)	0.16
Other city	160	15 (9.4%; 5.8%–14.9%)	9 (5.6%; 3.0%–10.3%)	136 (85.0%; 78.7%–89.7%)	138 (86.3%; 80.1%–90.7%)	
Residence place						
Urban	131	15 (11.5%; 7.0%–18.0%)	7 (5.3%; 2.6%–10.6%)	109 (83.2%; 75.9%–88.6%)	111 (84.7%; 77.6%–89.9%)	0.58
Rural	193	23 (11.9%; 8.1%–17.2%)	12 (6.2; 3.6%–10.6%)	158 (81.9%; 75.8%–86.7%)	159 (82.4%; 76.4%–87.1%)	
Education						
Junior	39	4 (10.3%; 4.1%–23.6%)	0 (0; 0–9.0%)	35 (89.7%; 76.4%–95.9%)	35 (89.7%; 76.4%–95.9%)	0.40
Senior	86	7 (8.1%; 4.0%–15.9%)	7 (8.1%; 4.0%–15.9%)	72 (83.7%; 74.5%–90.0%)	73 (84.9%; 75.8%–90.9%)	
College or higher	199	27 (13.6%; 9.5%–19.0%)	12 (6.0%; 3.5%–10.2%)	160 (80.4%; 74.3%–85.3%)	162 (81.4%; 75.4%–86.2%)	
Occupation						
Unemployed	86	7 (8.1%; 4.0%–15.9%)	7 (8.1%; 4.0%–15.9%)	72 (83.7%; 74.5%–90.0%)	73 (84.9%; 75.8%–90.9%)	0.65
Employed	238	31 (13.0%; 9.3%–17.9%)	12 (5.0%; 2.9%–8.6%)	195 (81.9%; 76.6%–86.3%)	197 (82.8%; 77.5%–87.0%)	

## Discussion

It is well known that CRS is a frequent cause of birth defects in those countries where rubella is endemic. It was a prominent problem in Asian region. To control rubella and prevent CRS, the WHO Regional Office for the Western Pacific (WPR) set a target for rubella incidence in 2012 year, which was less than 10 cases per million populations by 2015 [26]. Another important reason for preventing and controlling rubella was it has been an obstacle to eliminate measles [11, 27]. Measles and rubella are difficult to differentiate based on clinical symptom to each other in vaccine era. Laboratory testing for rubella, therefore, was recommended to be involved in measles surveillance system [12].

In the present study, the protective rate of anti-rubella IgG among pregnant women was 83.3%. It was similar with our previous investigation with smaller sample size, which revealed 84.5%/83.0% seroprevalence of rubella in 194 paired maternal/cord blood samples [28]. It is higher than that in another previous study among Chinese women of reproductive age, which reported that the prevalence of rate in women of reproductive age collected from 31 providences during 2010–2012 was 58.4%. In fact, there are significant regional differences on prevalence of rubella seropositivity (from 92.5 to 20.1%) [22]. However, this study was performed in different years and areas, and ELISA kits and cut off value used were probably different from what we used. De Paschale et al. [29] stressed that it is important to consider the guidelines used as an index of positivity. Adopting the cut off value of 10 IU/ml, our prevalence is comparable with the 85.8 and 87.5% seroprevalence reported in southern Italy [16] and Osogbo [23], respectively. However, it is lower than the prevalence recorded in Burkina Faso [17], Ontario [30], Haiti [18], and Jeddah [31] with seroprevalence of 93.3, 90, 92.8 and 91.6%, respectively.

The protective immunity was caused by infection or immunization. In China, rubella vaccine begin in 1990s, however, the vaccination coverage was low at that time because of self-supported and voluntary type in China before 2008. Zhou et al. reported that only 4.6% of women of reproductive age had a self-reported history [22]. However, 84.0% of pregnant women in 17–26 years group, 81.9% in 27–36 years group and 84.9% in 37–46 year group have protective immunity. Therefore, we deduced that this could be attributed to the prior exposure of the female to rubella virus which had conferred immunity before childbearing age. Another possibility is that there is still a sustained and frequent transmission of rubella virus in China. Therefore, systematic surveillance of CRS and continuous awareness schedule about the risk associated with rubella infection are needed.

Although the present rubella seroprevalence among pregnant women was relatively high, however, about 16.7% of pregnant women were susceptible to rubella. This absolute quantity is large considering the population of China. Despite the fact that both preconception and pregnancy screening for rubella are available, it is substantially underused in China. To control rubella and prevent CRS in China, preconception or pregnancy screening for rubella should be conducted irrespective of the history of rubella vaccination and clinical rubella. Then, vaccination of women of childbearing age susceptible to rubella or vaccination in the postpartum period should be strongly implemented. Although 60.5% (196/324) of women have undergone pregnancy, however, 16.3% of them are still susceptible to rubella. Therefore, these pregnant women’s general knowledge about rubella and CRS was poor. The government should take various means to improve the knowledge about the risk of rubella and CRS during pregnancy. The gynecologists and clinicians should exploit every opportunity to emphasize the importance of rubella screen and vaccination [16].

The strategy of adult women vaccination may prevent CRS but does not control rubella. Meanwhile, it still has a long way to implement 100% adult women vaccination. Therefore, a strategy of adolescent vaccination also should be considered to control rubella. As comprehensive measles elimination program had been established in China, rubella and CRS prevention programs may benefit from the established measles control campaigns [32]. It is more feasible to conduct an adolescent MR immunization program in students because of the mandatory education system in China [33]. The rationale for vaccinating adolescent is twofold - to prevent the spread of rubella and additionally to reduce the risk of CRS in the future.

## Conclusions

Despite the generally low vaccination coverage for rubella, most of Chinese pregnant women had potent rubella immunity. However, at least 16.7% of pregnant women were susceptible to rubella, which suggested rubella immunization in Chinese women at or before child-bearing age.

## Abbreviations

ACIP: Advisory Committee on Immunization Practices
CI: Confidence interval
CR: Congenital rubella syndrome
IgG: Immunoglobulin G
IU: International units
MMR: Measles-mumps-rubella
RCV: Rubella-containing vaccine
